# The Legion of Honor

**Published:** 1881-02

**Authors:** Ad. Lippe

**Affiliations:** Phila.


### THE LEGION OF HONOR.

BY AD. LIPPE, M.D., PHILA.

WHILE the American co-editor of *The Organon* was examining the December (1880) No. of *The Hahnemann Monthly*, looking for an answer to an oft repeated inquiry as to the nature of the accessory and supplementary principles to the law of the similars found and claimed to be in possession of the Hahnemann Club, the present owners of said journal, we found the following editorial.

"The energetic editors of *The Organon* have secured a list of names, comprising such homœopathic physicians as are willing to subscribe to a specified creed, *which has been derived from the principles of medicine as expounded by Hahnemann.** This list is denominated 'The Legion of Honor.' As a firm believer in Hahnemann's method of treating the sick, we added our name to the list when solicited. But in so doing, we claimed the right to use any external application which was employed in accordance with the law of the similars; and, further, we protested against some of the company we were called upon to keep. We recognized the names of quite a number in the Legion who we know are not practicing as they claim to. One name in the list before us is singularly out of place, since we know that its possessor has treated his patients with extravagant doses of crude drugs, and has used pounds of purgative medicine, and this, too, since signing the roll.

"If, then, *The Organon* wishes to publish a list of such practitioners as desire to *openly** express their allegiance to Hahnemanism, let there be a conscientious revision of the list as published. This can be done, because one of the editors has already been informed of several on the roll who are violating their contract. Either this, or drop the title of 'Legion of Honor.'"

* Italics are ours.

The chaos arising from different and contradictory definitions of homœopathy, induced some reflecting men in the profession to draw up a paper giving the essential points of the homœopathic doctrine; this "Declaration of Homœopathic Principles," was sent to such physicians as were known to practice homœopathy. Among others, one of the present editors of *The Hahnemannian Monthly* signed said declaration *without reserve*. We *now* find that he did so with the mental reservation to use any external application under the law of the similars, and we are further informed that he protested against some of the company he was called upon to keep. We are not aware of having seen or heard of any such protest. The learned editor may be sure of our most sincere sympathy, as there is nothing more humiliating than to be compelled to keep company with uncongenial persons. His request for a revision of the lists, comprising the names of the Legion of Honor, in order to dishonor any one who is practicing contrary to the homœopathic principles, is in itself very laudable, *but* the American co-editor of *The Organon* disclaims any positive knowledge of such a person or persons; true, he has been told by another member of the Legion of Honor that such persons exist, but no names were given nor was there any evidence offered to prove the correctness of such charges. There exists a maxim in law that every person shall be considered innocent till he has been proved to be guilty; furthermore, in our enlightened days, the accusor and accused are brought face to face and the onus of proof falls to the accuser. Hearsay testimony *even* if the informant enjoys the undisputed reputation for honest aims and strict veracity, is inadmissable. And as our learned friend appears to be very eager for a conscientious revision of the list as published, and as he *knows* at least one person who has violated his pledge grossly, it becomes his bounden duty to furnish us with the name (or names) and with positive proof to substantiate this accusation. Let him (or them) be tried before his peers and if found guilty his name (or their names) shall be dropped at once.

